# Supplementary material for: Spatial Transcriptomic Analysis Reveals Regional Transcript Changes in Early and Late Stages of rd1 Model Mice with Retinitis Pigmentosa
Source: Int J Mol Sci. 2023 Oct 3;24(19):14869. doi: 10.3390/ijms241914869 (PMC10573885; doi:10.3390/ijms241914869)
Supplement: Supplementary file 1 [file ijms-24-14869-s001.zip › Supplementary Figures.pdf]

**Supporting Information**

*Ying Zhou* [1]*, Yuqi Sheng* [1]*, Min Pan* [2]*, Jing Tu* [1]*, Yunfei Bai* [1]*, Xiangwei Zhao* [1]*, Qinyu Ge* [1*] *and Zuhong Lu* [1]

[1]State Key Laboratory of Bioelectronics, School of Biological Science & Medical Engineering, Southeast University, Nanjing 210096, China

[2]School of Medicine, Southeast University, Nanjing 210097, China

* Corresponding author: Professor Qinyu Ge, State Key Laboratory of Bioelectronics, School of Biological Science & Medical Engineering, Southeast University, Nanjing 210096, China. Email: geqinyu@seu.edu.cn

**Supplementary Figures:**

**Figure S1. Spatial sampling of sagittal sections of rd1 mice of different ages.**

**Figure S2. Comparison of retinal thickness between rd1 mice and control mice.**

**Figure S3. The analysis of the early and late stage of retinitis pigmentosa.**

**Figure S4. Venn diagram of differentially expressed genes at different times and locations.**

**Figure S5. The results of quantitative PCR of different retinal layers.**

**Figure S6. The results of quantitative PCR of different times.**

**Figure S7. KEGG mock-up of the close connection between phototransduction and VEGF pathway.**

**Figure S8. PPI diagram of the close relationship between phototransduction and VEGF pathway.**

**Figure S9. Immunofluorescence expression of EFNB2 protein in control C57BL/6J mice.**

**Figure S10. GO analysis of the pathways of the early and late stages of retinitis pigmentosa.**

**Figure S11. Expression trends of genes associated with neurodegenerative diseases over time.**

**Figure S12. Comparison of differences between the distal and proximal samples near the optic nerve.**

**Figure S13. Comparison of differences between the samples of P14 and P21.**

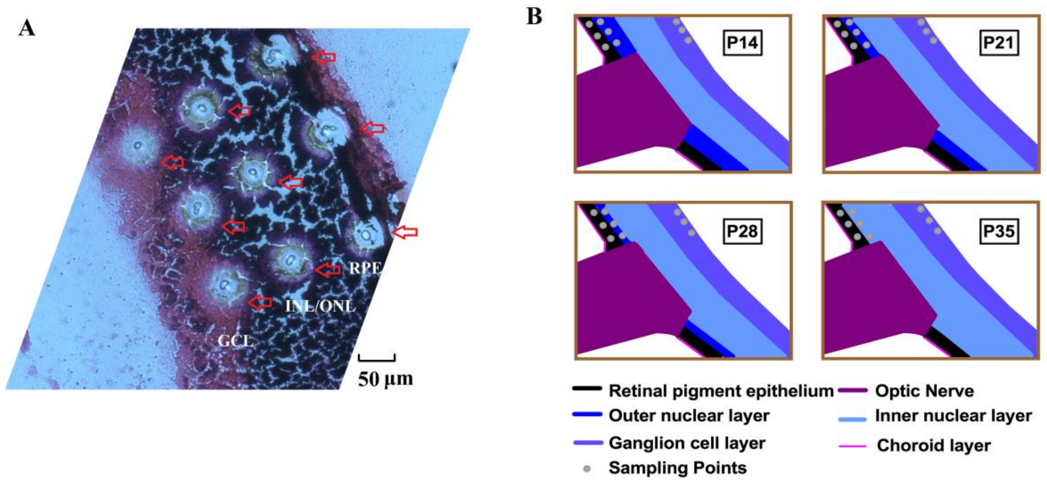


**Figure S1. Spatial sampling of sagittal sections of rd1 mice of different ages.**

**(A)** Micro-region samples of different retinal layers were obtained by micro-dissection. **(B)** First, the frozen sections required for sampling were selected through the optic nerve. Afterwards, the sampling was then divided into three groups including inner retinal layer (IRL), middle retinal layer (MRL) and outer retinal layer (ORL). The outer retina consists mainly of the retinal pigment epithelium (RPE) layer. The middle retina mainly includes the photoreceptor cell layer. Photoreceptor cell layer includes the inner nuclear layer (INL) and outer nuclear layer (ONL). The inner retina consists mainly of the ganglion cell layer (GCL). Finally, the samples were taken at different ages including P14, P21, P28 and P35.

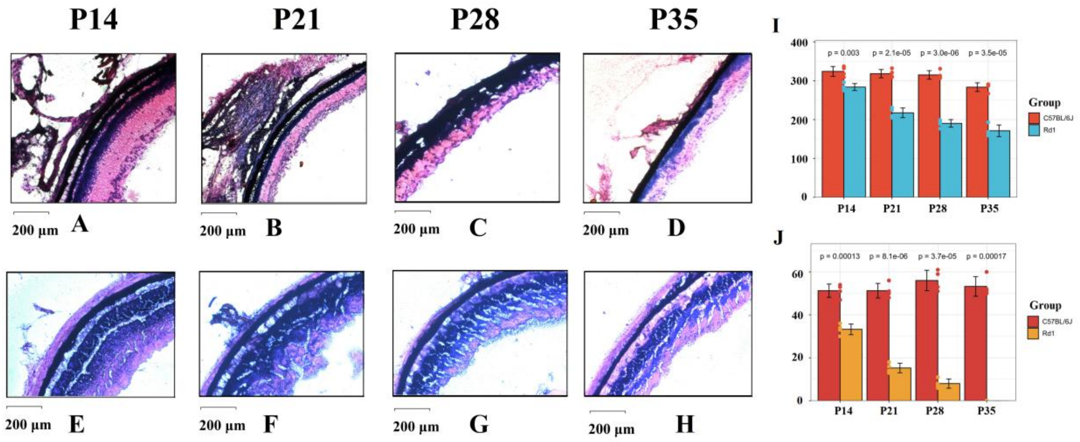


**Figure S2. Comparison of retinal thickness between rd1 mice and control mice.**

**(A-D)** Hematoxylin-eosin (HE) staining staining maps at four ages of P14, P21, P28, and P35 of rd1 mice. **(E-H)** HE staining maps at four ages of P14, P21, P28, and P35 of control C57BL/6J mice. **(I)** Total retinal thickness in sagittal sections between rd1 mice and control mice was compared at four ages. The retinal thickness of mice in the control group was about 300 μm while the retinal thickness of rd1 mice was thinner and decreased with age. The difference in the total retinal thickness between the two groups was statistically significant regardless of age ($P<0.01$). **(J)** In the control group, the thickness of outer nuclear layer was about 50 μm at four ages. The thickness of outer nuclear layer in rd1 mice became thinner with age and almost disappeared at P35. The difference in outer nuclear layer thickness between the two groups was statistically significant regardless of age ($P<0.001$).

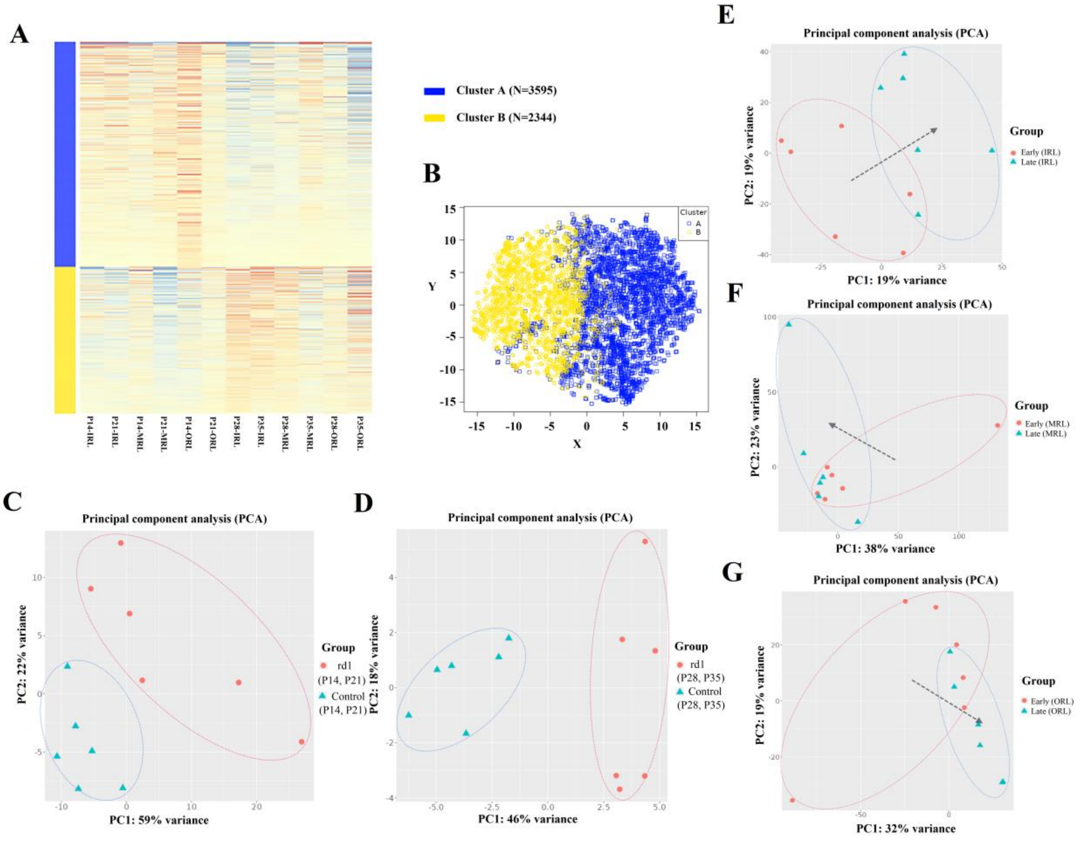

**Figure S3. The analysis of the early and late stage of retinitis pigmentosa.**

**(A)** The differentially expressed genes were grouped into Cluster A and Cluster B by k-means clustering method. The early (P14, P21) and late (P28, P35) group can be distinguished well. **(B)** Cluster A and Cluster B can be distinguished well. **(C)** The samples from the early and late stages were separated for PCA clustering. In the early stages (P14, P21), it was possible to separate rd1 and control by PCA cluster map. **(D)** In the late stages (P28, P35), it was possible to separate rd1 and control by PCA cluster map. **(E)** In the inner retinal layers (IRL), PCA clustering map of rd1 mice was able to demonstrate early and late trends. **(F)** In the middle retinal layers (MRL), PCA clustering map of rd1 mice was able to demonstrate early and late trends. **(G)** In the outer retinal layers (ORL), PCA clustering map of rd1 mice was able to demonstrate early and late trends.

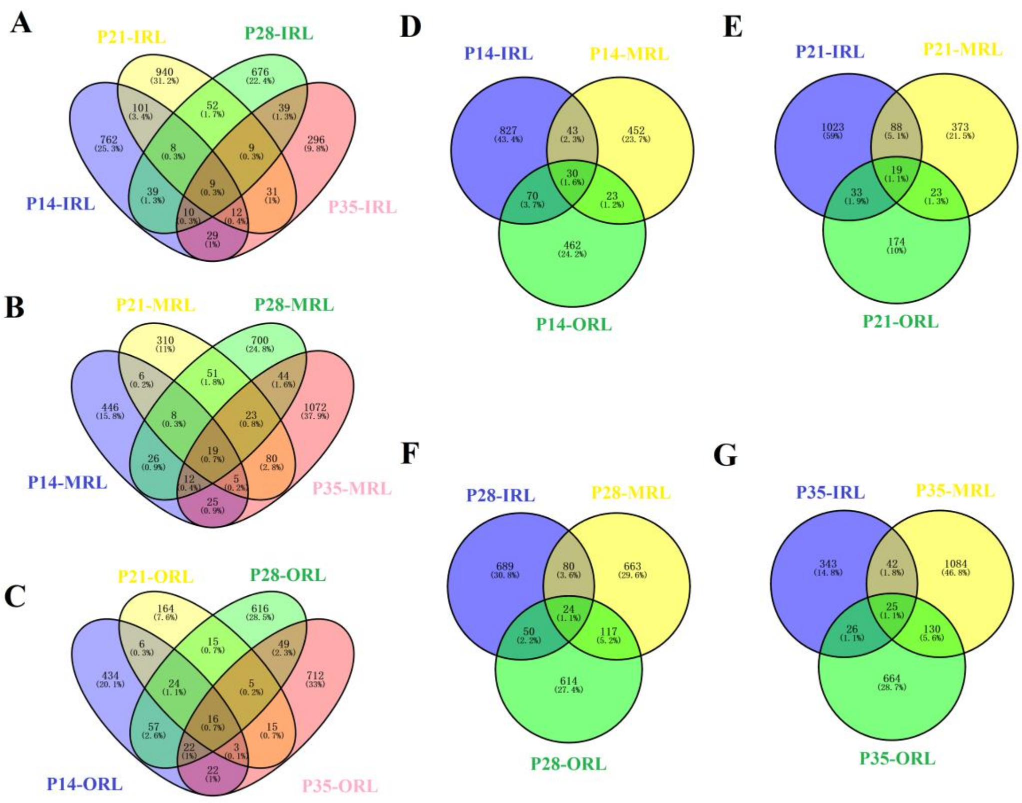


**Figure S4. Venn diagram of differentially expressed genes at different times and locations.**

**(A)** Venn diagram of IRL layer showed differentially expressed genes (DEGs) between rd1 and control group at four time points of P14, P21, P28 and P35. **(B)** Venn diagram of MRL layer showed DEGs between rd1 and control group at four time points. **(C)** Venn diagram of ORL layer showed DEGs between rd1 and control group at four time points. **(D)** Venn diagram of P14 time point showed DEGs between rd1 and control group in IRL layer, MRL layer and ORL layer. **(E)** Venn diagram of P21 time point showed DEGs between rd1 and control group in IRL layer, MRL layer and ORL layer. **(F)** Venn diagram of P28 time point showed DEGs between rd1 and control group in IRL layer, MRL layer and ORL layer. **(G)** Venn diagram of P35 time point showed DEGs between rd1 and control group in IRL layer, MRL layer and ORL layer.

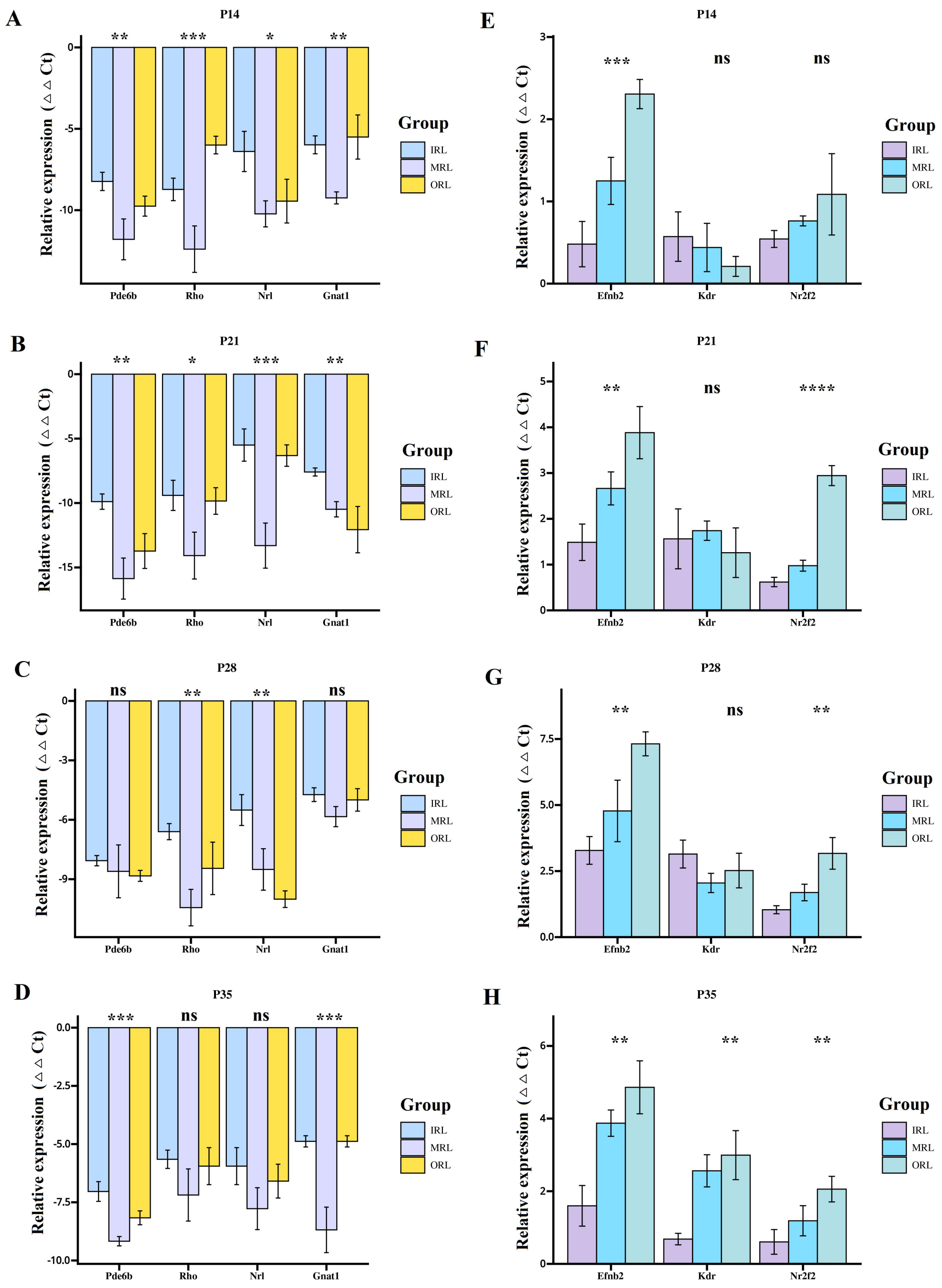


**Figure S5. The results of quantitative PCR of different retinal layers.**

(**A-D**) The results of quantitative PCR showed that the transcripts Rho, Pde6b, Gnat1 and Nrl were down-regulated in different retinal layers. (ns: no difference. *: difference, P<0.05. **: difference, P<0.01. ***: difference, P<0.001. ****: difference, P<0.0001.) (**E-H**) The quantitative PCR results of Efnb2, Kdr and Nr2f2 were down-regulated in different retinal layers and the differences between the different layers of Efnb2 were statistically significant.

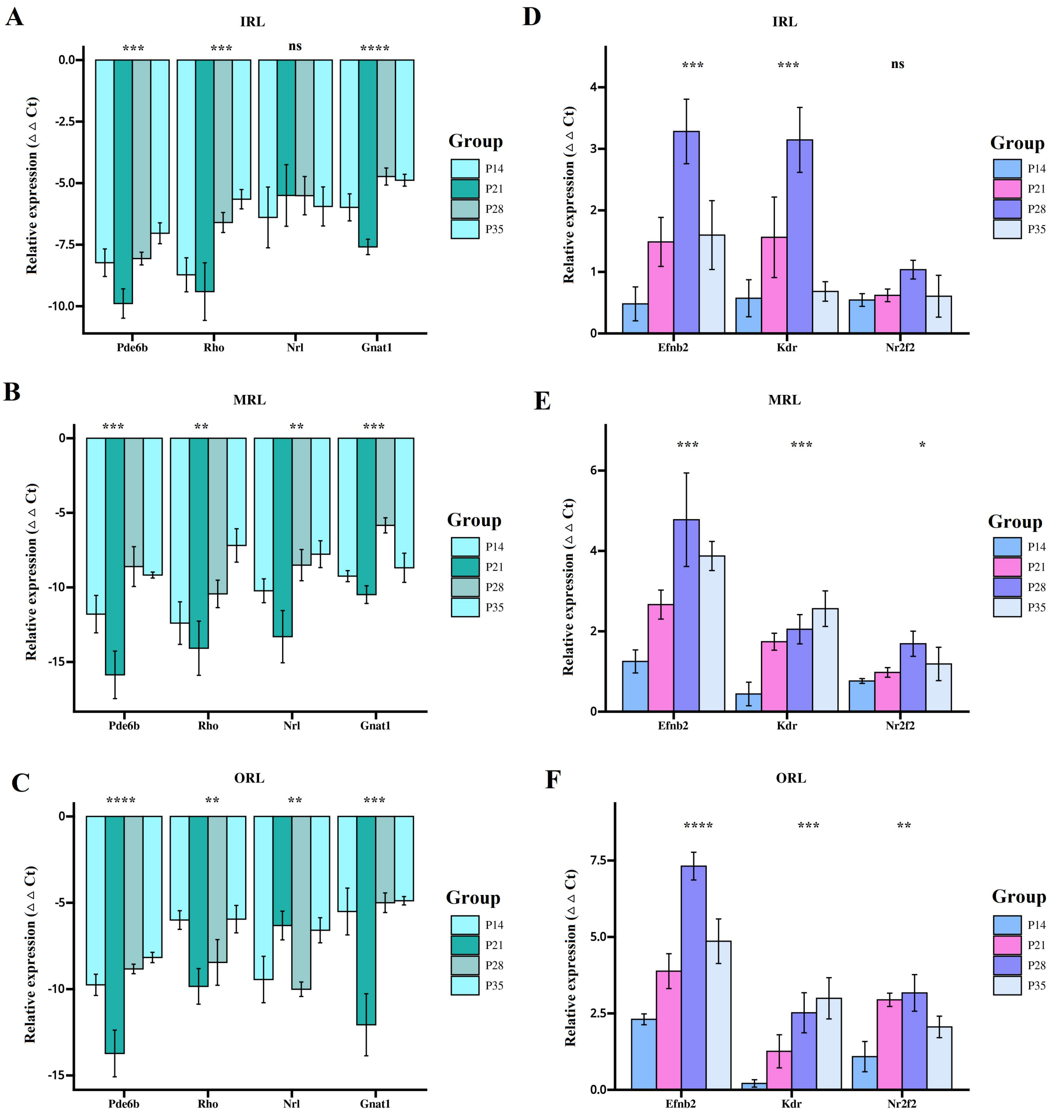


**Figure S6. The results of quantitative PCR of different times.**

(**A-C**) The results of quantitative PCR showed that Rho, Pde6b, Gnat1 and Nrl were down-regulated at all four times. (ns: no difference. *: difference, P<0.05. **: difference, P<0.01. ***: difference, P<0.001. ****: difference, P<0.0001.) (**D-F**) The quantitative PCR results of Efnb2, Kdr, and Nr2f2 showed up-regulation at four times, and the differences between different times of Efnb2 were statistically significant.

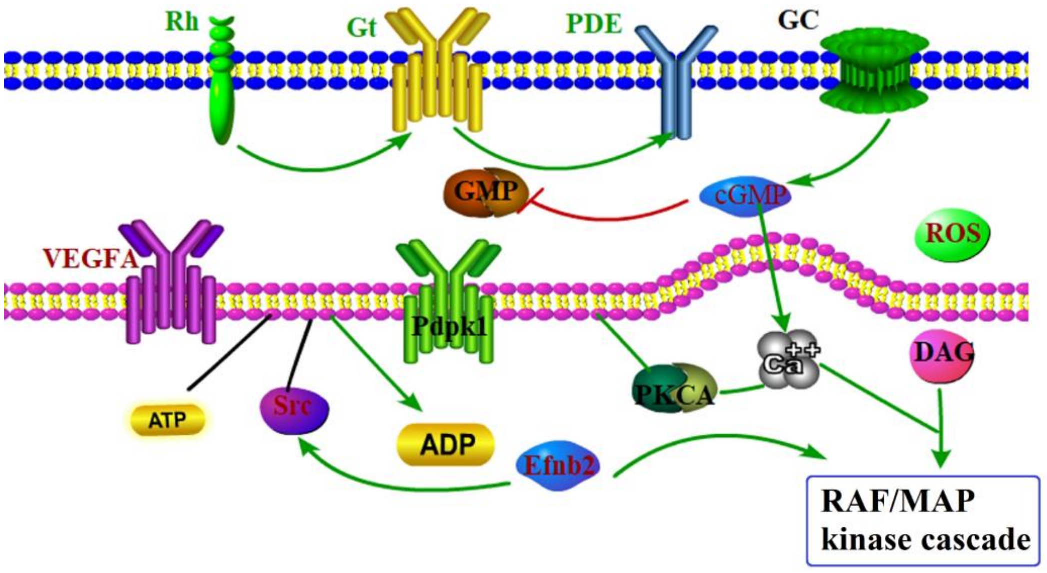


**Figure S7. KEGG mock-up of the close connection between phototransduction and VEGF pathway.**

In advanced retinal degeneration, a mutation in the Pde6b gene in rd1 mice results in reduced or complete loss of PDE enzyme activity. It leads to high concentrations of cGMP in the photoreceptor external segments persistently, accompanied by an increase in ROS. An increase in ROS will further lead to a decrease in retinal vascular network and blood perfusion in RP patients. After rod cell death, oxygen content in the outer retina is generally increased, and progressive oxidative damage occurs in both the retinal cone cells and the RPE layer. Reactive gliosis occurs in the retina and choroid with further oxidative damage and activation of retinal astrocytes. The blood-eye barrier is broken down in the late stages of retinal degeneration. With the activation of glial cells, the pro-inflammatory state increases and the antigen presentation increases. Elevated cGMPs may lead to over-activation of cyclic nucleotide-gated cations (CNGC) channels and protein kinase. $Ca^{2+}$ influx is mediated by cGMP activation, which in turn may lead to calpsin activation and photoreceptor cell death. Enhanced oxidative stress and $Ca^{2+}$ abnormalities associated with mitochondrial dysfunction further activate retinal and choroid remodeling mechanisms in RP. These pathways can create a positive feedback loop. Photoreceptor damage and the resulting oxidative stress may induce vascular changes through up-regulation of Efnb2. On the one hand, the up-regulation of Efnb2 gene in the VEGF pathway may act synergistically with Src gene to promote the migration of endothelial cell. On the other hand, Efnb2 will activate the MAPK signaling pathway to promote the proliferation of endothelial cell.

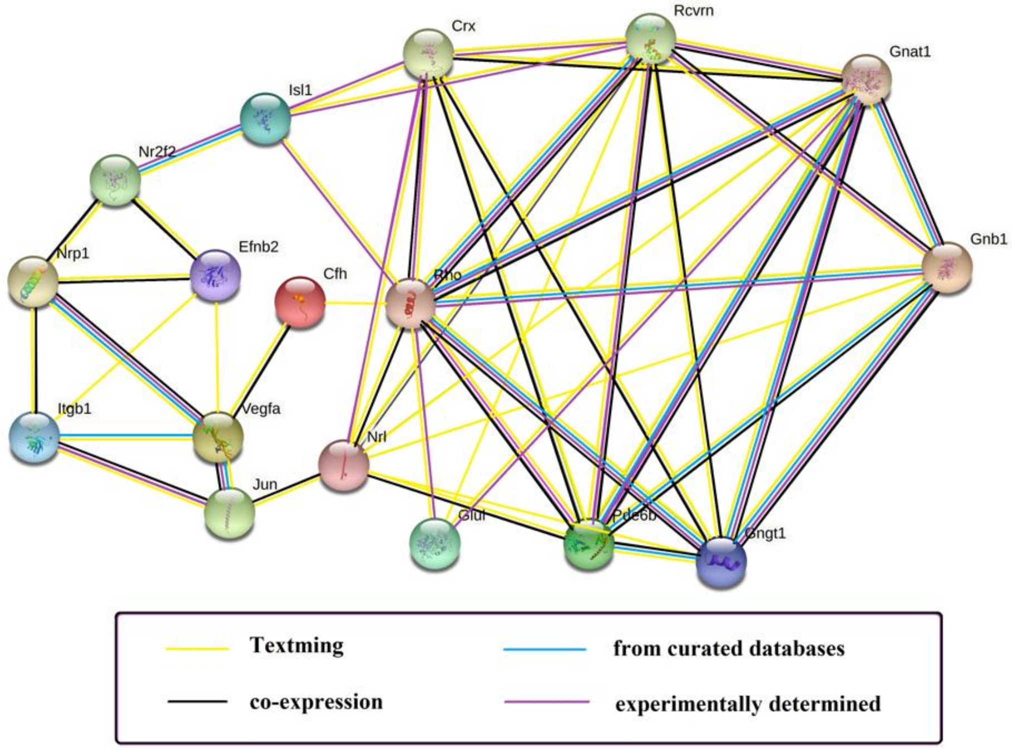


**Figure S8. PPI diagram of the close relationship between phototransduction and VEGF pathway.**

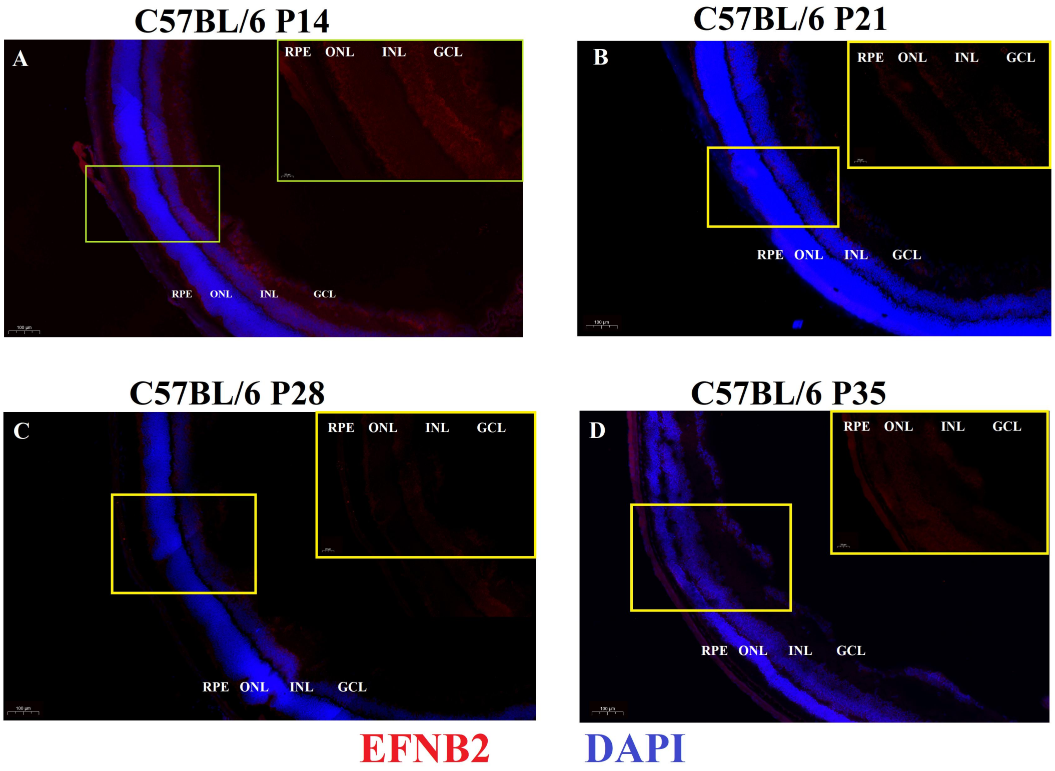


**Figure S9. Immunofluorescence expression of EFNB2 protein in normal C57BL/6J mice.**

**(A)** Immunofluorescence expression of EFNB2 protein of C57BL/6J mice in P14.

**(B)** Immunofluorescence expression of EFNB2 protein of C57BL/6J mice in P21.

**(C)** Immunofluorescence expression of EFNB2 protein of C57BL/6J mice in P28.

**(D)** Immunofluorescence expression of EFNB2 protein of C57BL/6J mice in P35.

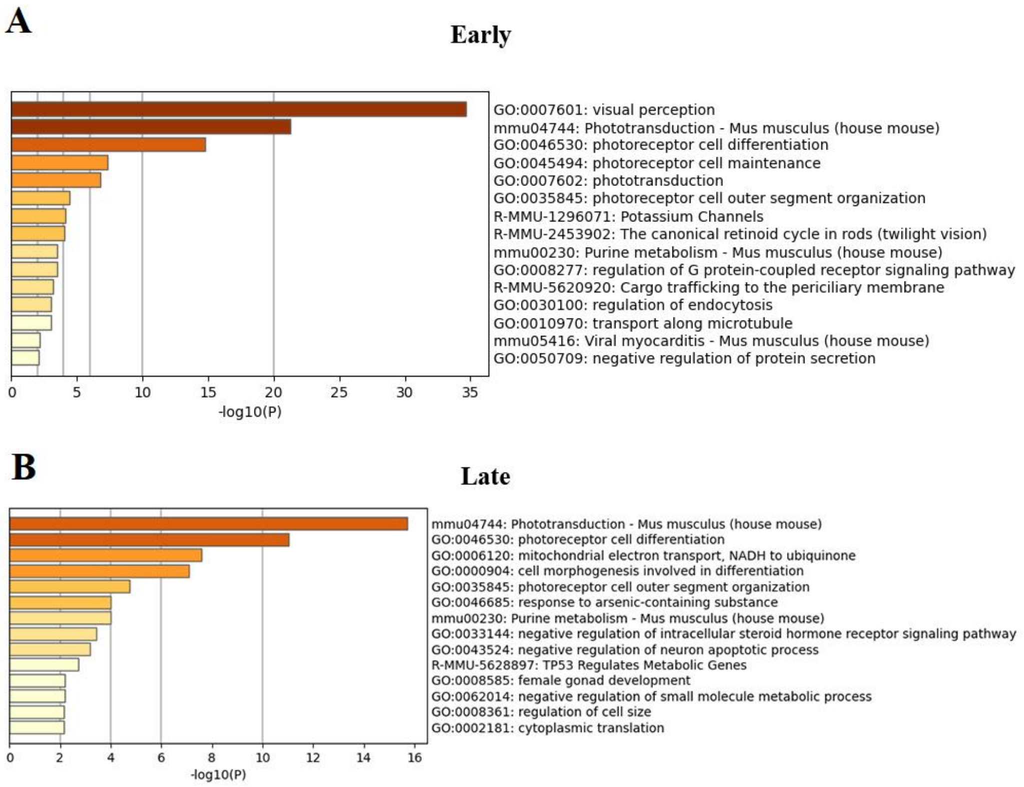


**Figure S10. GO analysis of the pathways of the early and late stages of retinitis pigmentosa.**

**(A)** The most prominent GO channels in the early stage of RP are visual perception, Phototransduction, photoreceptor cell differentiation, photoreceptor cell maintenance, phototransduction, photoreceptor cell outer segment organization, Potassium Channels, The canonical retinoid cycle in rods (twilight vision), Purine metabolism, regulation of G protein-coupled receptor signaling pathway, Cargo trafficking to the periciliary membrane, regulation of endocytosis, transport along microtubule, Viral myocarditis, negative regulation of protein secretion. **(B)** The most prominent GO channels in the late stage of RP are Phototransduction, photoreceptor cell differentiation, mitochondrial electron transport, NADH to ubiquinone,cell morphogenesis involved in differentiation, photoreceptor cell outer segment organization, response to arsenic-containing substance, Purine metabolism, negative regulation of intracellular steroid hormone receptor signaling pathway, negative regulation of neuron apoptotic process, TP53 Regulates Metabolic Genes, female gonad development, negative regulation of small molecule metabolic process, regulation of cell size, cytoplasmic translation.

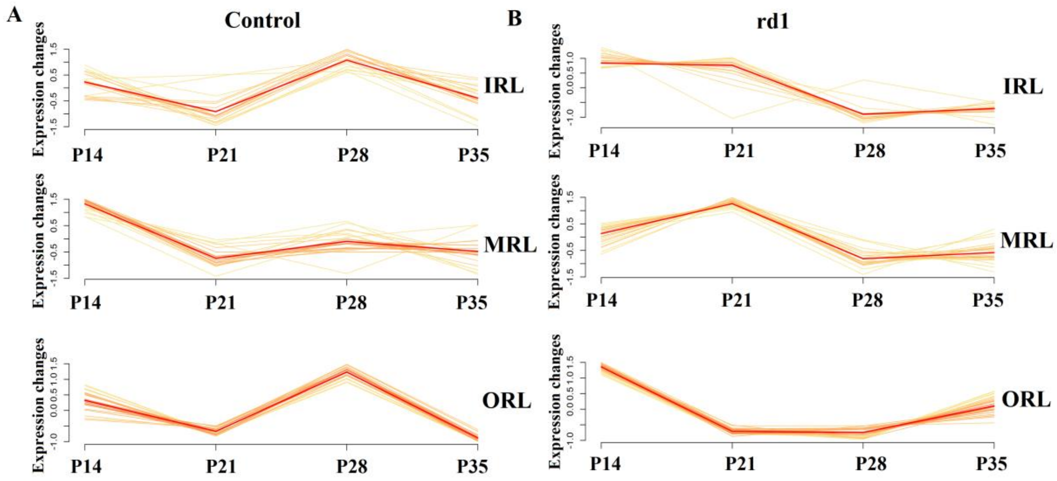


**Figure S11. Expression trends of genes associated with neurodegenerative diseases over time.**

Temporal dynamics of genes associated with neurodegenerative diseases from P14 to P35 in the IRL, MRL and ONL layers in the control group (**A**) is different from the rd1 group (**B**).

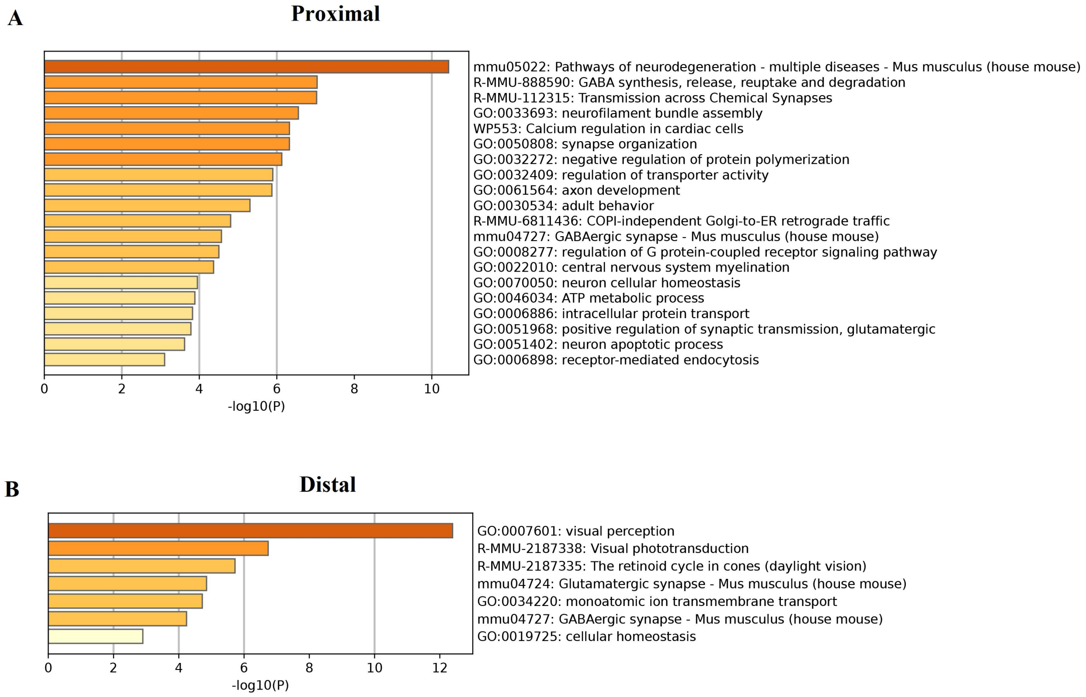


**Figure S12. Comparison of differences between the distal and proximal samples near the optic nerve.**

(**A**) Genes up-regulated in samples close to the proximal part of the optic nerve are mainly involved in Pathways of neurodegeneration-multiple diseases, receptor-mediated endocytosis, neuron apoptotic process, positive regulation of synaptic transmission, glutamatergic, intracellular protein transport, ATP metabolic process, neuron cellular homeostasis, central nervous system myelination, regulation of G protein-coupled receptor signaling pathway, COPI-independent Golgi-to-ER retrograde traffic, adult behavior, axon development, regulation of transporter activity, negative regulation of protein polymerization, synapse organization, Calcium regulation in cardiac cells, neurofilament bundle assembly, Transmission across Chemical Synapses, GABA synthesis, release, reuptake and degradation. (**B**) Genes up-regulated in samples near the distal part of the optic nerve are primarily involved in visual perception, cellular homeostasis, GABAergic synapse, monoatomic ion transmembrane transport, Glutamatergic synapse, The retinoid cycle in cones (daylight vision), Visual phototransduction.

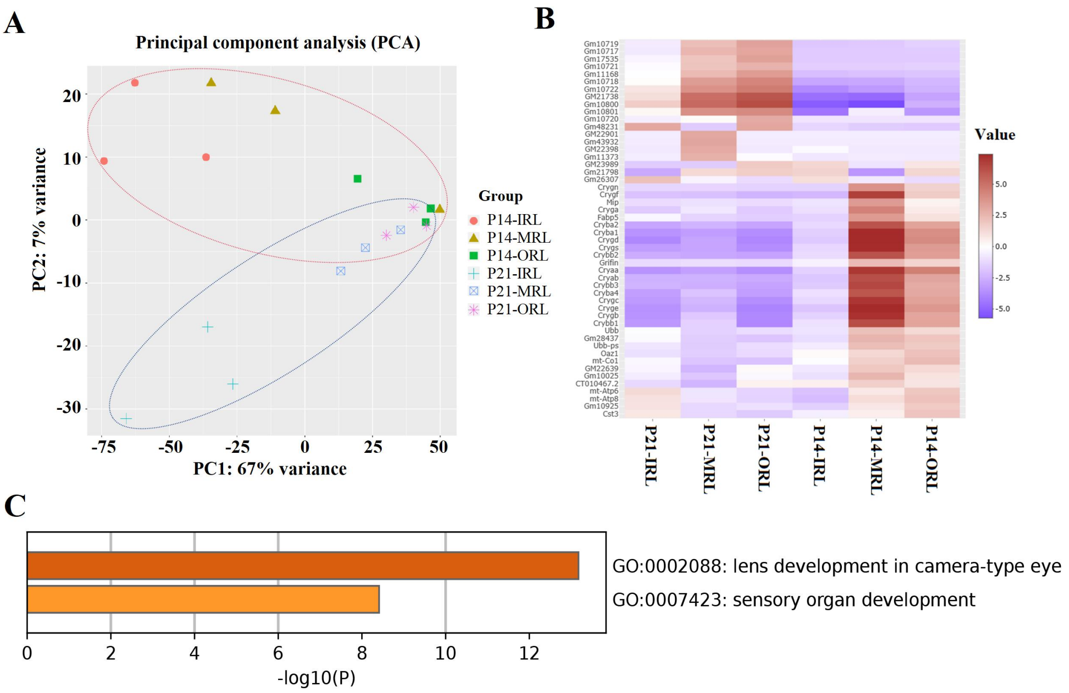


**Figure S13. Comparison of differences between the samples of P14 and P21.**

(**A**) PCA clustering plot distinguishes samples from P14 and P21. (**B**) The heat-map demonstrates the differential expression between P14 and P21. (**C**) GO analysis shows down-regulation of P21 in lens development in camera-type eye and sensory organ development.
